# Osteosarcopenic Visceral Obesity and Osteosarcopenic Subcutaneous Obesity, Two New Phenotypes of Sarcopenia: Prevalence, Metabolic Profile, and Risk Factors

**DOI:** 10.1155/2018/6147426

**Published:** 2018-05-16

**Authors:** Simone Perna, Daniele Spadaccini, Mara Nichetti, Ilaria Avanzato, Milena Anna Faliva, Mariangela Rondanelli

**Affiliations:** Department of Public Health, Experimental and Forensic Medicine, School of Medicine, Endocrinology and Nutrition Unit, University of Pavia, Pavia, Italy

## Abstract

**Background:**

The main criticism of the definition of “osteosarcopenic obesity” (OSO) is the lack of division between subcutaneous and visceral fat. This study describes the prevalence, metabolic profile, and risk factors of two new phenotypes of sarcopenia: osteosarcopenic visceral obesity (OSVAT) and osteosarcopenic subcutaneous obesity (OSSAT).

**Methods:**

A standardized geriatric assessment was performed by anthropometric and biochemical measures. Dual-energy X-ray absorptiometry (DXA) was used to assess body composition, visceral adipose tissue (VAT), subcutaneous adipose tissue (SAT), osteoporosis, and sarcopenia.

**Results:**

A sample of 801 subjects were assessed (247 men; 554 women). The prevalence of osteosarcopenic obesity (OSO) was 6.79%; OSSAT and OSOVAT were, respectively, 2.22% and 4.56%. OSVAT (versus the others) showed a higher level of inflammation (CRP and ESR, *p* < 0.05), bilirubin (*p* < 0.05), and risk of fractures (FRAX index over 15%, *p* < 0.001). Subjects with OSSAT did not show any significant risk factors associated to obesity.

**Conclusions:**

The osteosarcopenic visceral obesity phenotype (OSVAT) seems to be associated with a higher risk of fractures, inflammation, and a worse metabolic profile. These conditions in OSVAT cohort are associated with an increase of visceral adipose tissue, while patients with OSSAT seem to benefit related to the “obesity paradox”.

## 1. Introduction

The main criticism of the definition of osteosarcopenic obesity or sarcopenic obesity is due to a lack of division between subcutaneous or visceral fat mass. Osteosarcopenic obesity (OSO) is a multifactorial syndrome that includes the following conditions: decrease of muscle mass and bone (osteopenia/osteoporosis and sarcopenia) and an increase of adiposity (obesity) [1]. Regarding the condition of adiposity, related to sarcopenia and osteoporosis, until now no study has considered the role of visceral fat.

Recent evidence by Dimitri et al. suggests that site-specific adiposity may exert differing effects on bone “with visceral fat acting as a pathogenic fat depot and subcutaneous fat exerting protective effects” [2]. For example, in the study performed by Perna et al., a positive association between adiposity and BMD was explained by biomechanical forces or by increased aromatization of androgens to weak estrogens in subcutaneous fat tissue [3].

The main risk factors associated with OSO are poor nutritional status, a high grade of inflammation, and fractures [4]. Recent findings suggest that the incidence of sarcopenia, sarcopenic obesity, and osteosarcopenic obesity was 31.5, 5.1, and 4.1%, respectively [5]. Recently, the prevalence of OSO was assessed as 19% in a study performed in Mexico. In addition, OSO is a common condition in middle-aged and older women, and it is independently associated with frailty and poor physical performance [6].

There are no other studies that have assessed the prevalence of OSO in a large population cohort and specifically none in Europe. As shown recently, adiposity over 33% is negatively associated with bone mineral density (BMD) [7]. However, obesity has a role in overnutrition, as a mediator of the adverse effect in osteoporosis and sarcopenia pathogenesis [8].

In recent years, there has been increasing interest in the influence of visceral (VAT) and subcutaneous adipose tissue (SAT) on sarcopenic patient outcomes. Obesity has been identified as an adverse factor in sarcopenia [9]. As a matter of fact, no studies show the effects of visceral or subcutaneous fat as adverse prognostic factors in sarcopenic patients.

DXA quantification of fat distribution is a potentially valuable tool to diagnose these situations. To our knowledge, only one study has examined the relationship between visceral adipose tissue and muscle mass, reported to be a principal determinant of major morbidity in patients undergoing pancreaticoduodenectomy for cancer [10]. Most mechanisms of sarcopenia are also associated with visceral obesity, which may lead to a vicious cycle of intricate interactions among risk factors. Insulin resistance plays an important role in muscle fiber atrophy and mitochondrial dysfunction [11].

Also, intermuscular adipose tissue (IMAT) has been observed in the skeletal muscles of older adults with sarcopenia [12]. The relationship between the possible negative outcome associated with visceral adipose tissue and muscle mass is unclear.

This study describes for the first time in the literature two different phenotypes of sarcopenia: osteosarcopenic visceral obesity (OSVAT) and osteosarcopenic subcutaneous obesity (OSSAT), in addition to analyzing the prevalence, metabolic profile, and the risk factors.

## 2. Methods

### 2.1. Study Population

This cross-sectional study in elderly patients was performed in the city of Pavia (Italy). Inclusion criteria were (1) admission to the post-acute geriatric care unit for functional loss secondary to a nondisabling medical disease; (2) aged 65 years or older; (3) bedridden patients who were ambulatory prior to hospitalization; and (4) willingness to participate and to provide signed informed consent. At time of admission, the patients were not diagnosed with disabling diseases that could directly affect muscle weakness (such as neurological diseases, hip fractures, or amputations). However, participants with diabetes, metabolic disease, or neoplasia, as well as patients treated with steroids, or who were able to walk, were excluded.

Exclusion criteria were subjects affected by acute illness; severe liver (as defined by ESPEN guidelines) [13], heart (European Society of Cardiology proposed guidelines for the diagnosis) [14], or kidney dysfunction (acute kidney “risk, injury, failure” as defined by the newly developed RIFLE classification) [15]; or severe dementia (MMSE < 18 points) [15].

The stability of BMI is fundamental in our study because the body weight represents the primary difference between three groups; for this reason, we considered only people who had a stable clinical situation for the previous six months. The data were collected over a six-year period, from January 2011 to January 2017, in collaboration with the University of Pavia. All participants gave informed consent, and the research institute ethics committee approved the study.

### 2.2. Observed Variables

As suggested by Ilich et al. [5], there are two assessment steps for obtaining a more comprehensive diagnosis for OSO. This could be performed in any clinical setting with the DXA technology. Thus, the physical diagnosis would range from osteopenia, sarcopenia, and/or obesity to osteopenic obesity, sarcopenic obesity, osteopenic sarcopenia, and osteosarcopenic obesity (Table 1).

### 2.3. Diagnosis of Osteosarcopenic Visceral\Subcutaneous Obesity

Diagnostic criteria for osteosarcopenic obesity based on body composition (via dual-energy X-ray absorptiometry (DXA)) are shown in Table 1 [4, 5].

### 2.4. Body Composition Assessment

Body composition such as free fat mass (FFM), fat mass (FM), gynoid and android (subcutaneous or visceral) fat distribution was measured with dual-energy X-ray absorptiometry (DXA) using a Lunar Prodigy DXA (GE Medical Systems). The in vivo CVs were 0.89% and 0.48% for whole-body fat (fat mass) and FFM, respectively.

### 2.5. Diagnosis of Sarcopenia

The skeletal muscle index (SMI) was taken as the sum of the fat-free soft tissue mass of arms and the fat-free soft tissue mass of legs and dividing by height squared. Whole body and FFM were divided by height squared to obtain the FFM index (FFMI). FFM depletion was defined as having whole body FFMI below the 5th centile for age- and gender-matched healthy subjects [4, 5, 16]. Table 1 summarises the main phenotype of sarcopenia following the classification by Perna and Rondanelli and following the preexisting classification by Ilich et al. [4, 5].

### 2.6. Diagnosis of General Obesity

Obesity was diagnosed as fat mass over 38% in women and 27% in men. Taking into account that fat mass in normal weight subjects corresponds to 15% in men and 25% in women, ideal lean body mass was calculated in kilogram as the sum of 85% of ideal body weight in men or 75% in women plus 25% of excess weight, expressed as body weight exceeding a reference body weight corresponding to a BMI > 25 kg/m^2^, considering that excess body weight includes not only fat mass but also a certain amount of muscle mass [5–19].

### 2.7. Diagnosis of Visceral or Subcutaneous Obesity

Abdominal subcutaneous fat (SAT) and visceral fat (VAT) were estimated within the android region. Fat mass data from DXA was transformed into X-ray computed tomography (CT) adipose tissue volume using a constant correction factor (0.94 g/cm^3^). This gave the VAT/SAT ratio. Subjects with values over >1 were classified as having visceral obesity, and subjects with values under <1 were classified as having subcutaneous obesity.

### 2.8. Assessment of Bone Mineral Density

Bone mineral density (BMD) (g/cm^2^) of the total hip was measured using DXA. BMD was labeled as normal when *T*-score > −1.0, osteopenic if *T*-score < −1.0, and osteoporosis when *T*-score ≤ −2.5 [19]. In addition, we evaluated the FRAX index that considers bone mineral density (BMD) at the femoral neck and the osteoporosis risk factors to calculate the fracture risk at 10 years [20].

### 2.9. Blood Sample Measurements

Fasting venous blood samples were drawn between 8 am and 10 am, with the subjects in a sitting position. Blood handling and collection were carried out under strictly standardized conditions. Folate and vitamin B12 were determined using an immunoassay, and high-performance liquid chromatography was used to measure total plasma homocysteine levels. Serum albumin was also analyzed using a nephelometric method, with a 2% coefficient of variation. Fasting blood total cholesterol and triglyceride levels were measured by automatic biochemical analyzer. High-sensitivity C-reactive protein (CRP), erythrocyte sedimentation rate (ESR), creatinine, azotemia, glycemia, and complete hemochrome were also assessed.

### 2.10. Assessment of Functional Performance

Handgrip strength was assessed using a Jamar dynamometer adhering to the standardized protocol recommended by the American Society of Hand Therapists. Dominant and nondominant handgrip strength was measured with a calibrated dynamometer (Baseline, Elmsford, NY, USA). The grip handle was adjusted to accommodate the size and comfort of the participant's hand, and the elbow was flexed to 90° to guarantee the strongest grip strength measurement. A weak handgrip was defined as <30  kg for men and <20  kg for women using the average value of the two handgrip measurements of the dominant hand [21–23].

### 2.11. Assessment of Hydration

All subjects underwent bioelectrical impedance vector analysis (BIVA) with a single-frequency bioimpedance analyzer (Model BIA 101, AKERN-RJL, Italy). Measurements were performed while the subjects lay comfortably, with the limbs abducted from the body. Body hydration was determined by injecting 800 *µ*A and 50 kHz alternating sinusoidal current using a standard tetrapolar technique [24, 25].

### 2.12. Statistical Analysis

All analyses were performed using Statistical Package for the Social Sciences, version 22.0 (SPSS Inc., Chicago, IL, USA). Descriptive statistics representing raw data for each category and the full sample were provided, including means, standard deviations, and frequencies, where appropriate.

After the verification of the normal distribution of the continuous variables, data were analyzed and statistically compared between groups using one-way ANOVA. Variances were considered to be statistically significant for *p* value < 0.05.

Pearson's correlation analysis was used to compare the association between (OSVAT and OSSAT) versus other outcomes. The model was adjusted by the covariates of age and sex.

## 3. Results

### 3.1. Sample

**Figure 1:**
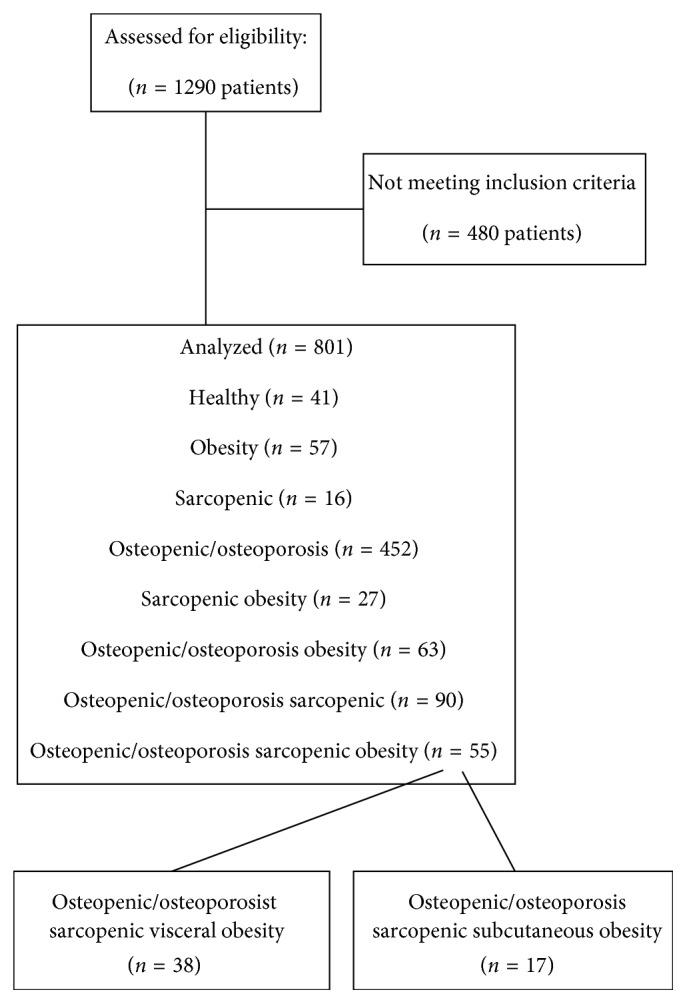
Flow diagram of the study.

As shown in Figure 1, we selected 1290 patients: 480 patients were lost because they were unable to be assessed with DXA. The sample of 801 subjects was categorized as follows: healthy (*n*=41), with obesity (*n*=57), with sarcopenia (*n*=16), with osteopenia or osteoporosis only (*n*=442), with sarcopenic (visceral or subcutaneous) obesity (*n*=27), with osteopenic or osteoporosis and obesity (*n*=63), with osteopenia or osteoporosis and sarcopenic (*n*=90), and with osteosarcopenic obesity (*n*=55).

### 3.2. Prevalence of Osteosarcopenic SAT and VAT

The prevalence of osteosarcopenic obesity was 6.86%, where the OSOSAT and OSOVAT prevalence was, respectively, 2.10% and 4.70%.

It is interesting that those who were healthy (without sarcopenia, obesity, and osteopenia or osteoporosis) were only 5.5% of the sample.

The elderly with osteopenia and osteoporosis alone made up over 60% of the sample. This points the attention to this condition that involves 69% of women and 37% of men (without sarcopenia and obesity). In addition, sarcopenia is closely to osteoporosis and obesity.

### 3.3. Metabolic Profile of OS Visceral Obesity

**Figure 2:**
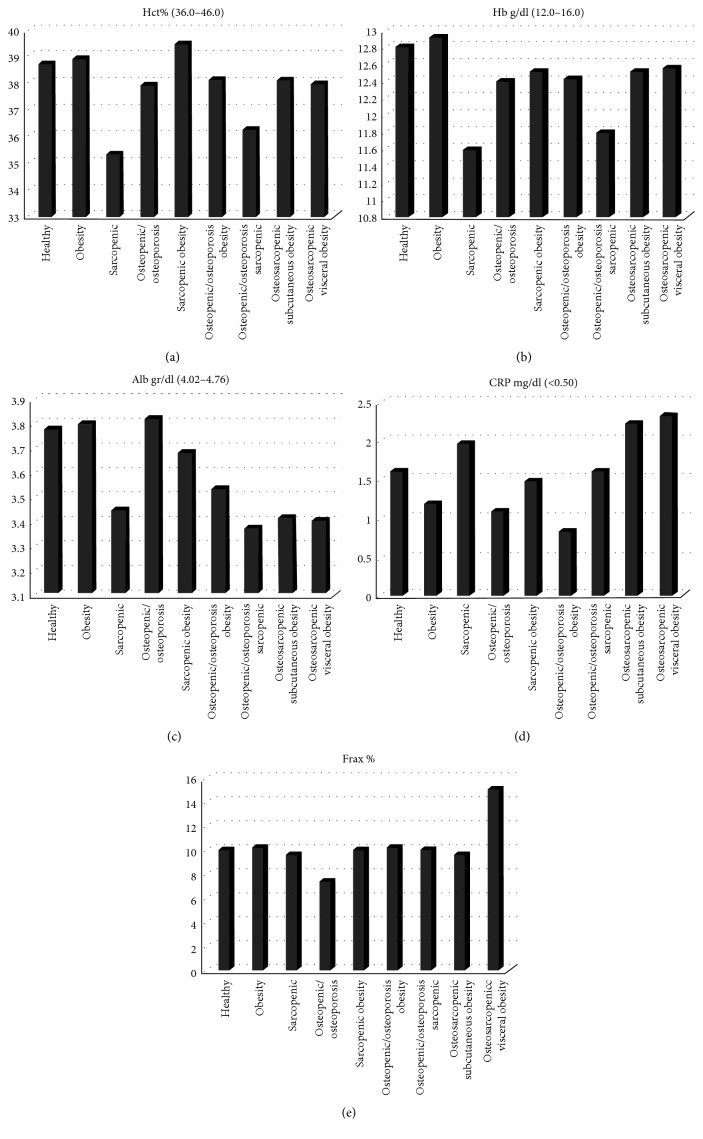
Metabolic outcomes and risk of fracture in the sample.

Table 2 describes the characteristics of the sample. One-way ANOVA analysis of variance detected statistically significant differences between groups (*p* < 0.05) in hemoglobin, erythrocyte, iron, triglyceride, cholesterol, erythrocyte sedimentation rate, and FRAX index. As shown in Figure 2, OSVAT (versus the other cohorts) showed a higher level of inflammation (CRP > 2.34 mg/dl), higher risk of fracture (FRAX > 15%), and a reduction of glycemic control (glycemia > 112 mg/dl). In addition, VAT in OSVAT was significantly correlated to FRAX (*r* = 0.316; *p* < 0.05). We found that osteosarcopenic visceral obesity subjects had albumin values under the normal range (<4 gr\dl) and a higher level of azotemia (>40 mg\dl), glycemia (>110 mg\dl), and FRAX (15% of risk of fractures). Also, the level of strength was under the cut-off level of 20 kg for women.

### 3.4. Association between OSSAT\OSVAT with All Other Outcomes

Table 3 reports the results of Pearson's correlation analysis of VAT and SAT with other metabolic outcomes in the cohort of osteosarcopenic obesity women. VAT was significantly correlated to FRAX index (*r* = 0.316; *p* < 0.05) and to bilirubin (*r* = 0.328; *p* < 0.05). SAT was significantly correlated to platelet (*r* = 0.290; *p* < 0.05) and creatinine (*r* = −0.299; *p* < 0.05). No other correlations were observed.

## 4. Discussion

For the first time in the literature, this study describes two new phenotypes of sarcopenia: osteosarcopenic visceral obesity and osteosarcopenic subcutaneous obesity.

This study has been performed using dual-energy X-ray absorptiometry (DXA), with a new tool to quantify visceral adipose tissue (VAT) in the android region. In this way, the fat in the android region was categorized as visceral or subcutaneous. Using VAT, and in particular the VAT/SAT ratio with a cut-off ≥1 by DXA, we defined the visceral or subcutaneous phenotype.

An important finding of this study was the prevalence of OSVAT (4.56%) compared to OSSAT (2.22%). In general, the prevalence of OSO was 6.79%.

The main finding of this study is that VAT is deleterious for bones. In fact, the elderly patients with OSVAT had a higher risk of fractures, as suggested by a higher FRAX index.

The literature supports our results as regards the negative effects on bone of visceral adipose tissue. In particular, the negative effects are related to a proinflammatory state that promotes bone resorption [25–27].

Until now, there was great confusion in the literature of the effects on bone of obesity. We know that on one side obesity in elderly may be protective, following “obesity paradox,” while on the other side fat heavily decreases osteoblastogenesis [27].

Similar findings were highlight by Ayça and Ilich et al. [28, 29], who showed that increased visceral fat and lower handgrip strength may be related to increased no-reflow rate.

In addition, we found differences on several metabolic outcome, such as hemoglobin, triglycerides, iron, calcium, and cholesterol (as indicators of nutritional status and mortality risk). Although OSVAT showed an impairment of nutritional status, as suggested by the level of albumin under the normal range with an increase of functional decline, with lower handgrip strength, this data did not differ from OSSAT.

Limitations to our study include small sample size of OSO and a majority of the sample being women. We could only study causation in the women in this study due to lack of men with these diagnoses. This study lays the foundations for the diagnostic values of these phenotypes of sarcopenia. We highlight that the current cut-point for visceral/subcutaneous obesity was defined using the VAT/SAT ratio. No study has assessed this specific cut-off.

## 5. Conclusion

Osteosarcopenic visceral obesity phenotype (OSVAT) seems to be closely linked to a higher risk of fractures, inflammation, and a worse metabolic profile. These conditions in the OSVAT cohorts are associated with an increase of visceral adipose tissue. It is important that patients with OSVAT must be assessed and identified in the clinical setting, because they are a cohort with a major exposure of risks. In contrast, the patients with OSSAT seem to be beneficiaries of the obesity paradox.

## Figures and Tables

**Table 1:** Diagnostic criteria for osteosarcopenic obesity based on body composition (via dual-energy X-ray absorptiometry) [2, 3, 5].

Diagnostic criteria	*T*-score for BMD ≤ −1.0 SD at the hip femoral	SMI > 5.5 kg/h^2^ for women and 7.23 kg/h^2^ for men	Fat mass ≥ 38% for women and ≥28% for men	Visceral/subcutaneous fat ratio > 1
Osteopenia/osteoporosis	Yes	No	No	No
Sarcopenia	No	Yes	No	No
Visceral obesity	No	No	Yes	Yes
Subcutaneous obesity	No	No	Yes	No
Osteopenic sarcopenia	Yes	Yes	No	No
Osteopenic subcutaneous obesity	Yes	No	Yes	No
Osteopenic visceral obesity	Yes	No	Yes	Yes
Sarcopenic subcutaneous obesity	No	Yes	Yes	No
Sarcopenic visceral obesity	No	Yes	Yes	Yes
Osteosarcopenic subcutaneous obesity	Yes	Yes	Yes	No
Osteosarcopenic visceral obesity	Yes	Yes	Yes	Yes

BMD: bone mineral density; SMI: skeletal muscle index [5].

**Table 2:** Characteristics and metabolic outcomes evaluated by ANOVA analysis.

Variables	Healthy (*n = *41)	Obesity (*n = *57)	Sarcopenic (*n *=* *16)	Osteopenic/osteoporosis (*n *=* *452)	Sarcopenic obesity (*n *=* *27)	Osteopenic/osteoporosis obesity (*n *=* *63)	Osteopenic/osteoporosis sarcopenic (*n *=* *90)	Osteosarcopenic subcutaneous obesity (*n = *17)	Osteosarcopenic visceral obesity (*n = *38)	*p* value
*General data*										
Age (years)	78.00 (9.25)	77.11 (7.88)	75.94 (7.41)	81.38 (7.19)	77.04 (9.59)	81.00 (2.65)	82.29 (7.00)	81.32 (4.32)	**82.43 (3.44)**	*p * **<0** **.001**
*Biochemical parameters*										
WBC K/*µ*L (4.00–10.00)	6.60 (1.85)	7.25 (2.29)	8.12 (2.74)	6.86 (3.42)	8.12 (2.34)	8.05 (2.31)	7.23 (2.56)	8.03 (2.18)	8.24 (1.34)	0.059
RBC M/*µ*L (4.00–5.00)	4.32 (0.49)	4.30 (0.62)	3.97 (0.89)	4.21 (0.71)	4.33 (0.61)	4.05 (0.30)	4.12 (0.65)	4.16 (0.45)	4.15 (0.49)	0.545
Hb g/dl (12.0–16.0)	12.81 (1.77)	12.92 (1.60)	11.60 (2.04)	12.41 (1.69)	12.52 (1.44)	12.43 (1.53)	11.80 (1.87)	12.52 (1.31)	12.56 (1.41)	*p * **<0** **.001**
Hct % (36.0–46.0)	38.74 (4.98)	38.91 (4.71)	35.38 (6.84)	37.91 (4.80)	39.46 (4.14)	38.10 (3.08)	36.23 (5.34)	38.08 (1.31)	37.95 (4.23)	*p * **<0** **.001**
MCV fL (81.0–93.0)	88.93 (7.65)	88.56 (6.85)	91.83 (1.62)	91.47 (7.82)	87.95 (5.34)	91.90 (1.84)	81.17 (14.34)	90.06 (3.64)	84.867 ± 6.0335	0.103
PLT K/*µ*L (140–450)	253.28 (99.03)	245.19 (87.80)	303.70 (88.56)	241.51 (99.03)	257.45 (79.50)	216.67 (61.01)	242.01 (82.83)	292.84 (101.03)	234.703 ± 71.5732	0.418
Lymph *n* K/*µ*L (0.80–3.60)	1.73 (0.59)	1.98 (0.53)	1.46 (0.52)	1.78 (0.73)	2.12 (0.43)	1.75 (0.31)	1.71 (0.57)	2.10 (0.55)	1.72 (0.58)	0.289
Iron mcg/dl (45–145)	65.19 (32.29)	73.67 (26.63)	47.36 (25.06)	68.46 (34.28)	57.96 (26.90)	69.33 (34.27)	57.78 (32.01)	68.62 (37.12)	61.324 (25.41)	*p * **<0** **.001**
Trg mmol/L (<200)	110.78 (43.59)	141.29 (68.36)	122.00 (38.55)	121.28 (60.56)	136.33 (42.81)	100.00 (19.98)	102.95 (38.29)	136.58 (46.91)	122.58 (47.19)	*p * **<0** **.05**
Total Chol mmol/L (<200)	184.58 (41.04)	195.68 (44.50)	154.80 (32.59)	189.46 (43.10)	187.04 (45.07)	120.00 (33.18)	174.39 (45.46)	190.00 (37.59)	166.38 (48.56)	*p * **<0** **.001**
Alb g/dl (4.02–4.76)	3.77 (0.54)	3.79 (0.46)	3.44 (0.60)	3.81 (2.99)	3.67 (0.53)	3.53 (0.42)	3.36 (0.57)	3.40 (0.35)	3.40 (0.53)	0.787
Creat mg/dl (0.57–1.11)	0.86 (0.28)	0.91 (0.28)	0.98 (0.50)	1.04 (2.61)	1.15 (0.67)	1.47 (0.55)	0.95 (0.62)	0.80 (0.18)	1.07 (0.70)	0.998
Azotemia mg/dl (14–40)	39.16 (13.10)	41.41 (16.90)	39.57 (16.39)	45.31 (22.94)	47.54 (24.17)	73.67 (31.90)	47.58 (34.35)	37.23 (12.66)	49.76 (24.84)	0.193
Na mmol/l (136–145)	139.11 (3.39)	139.06 (3.40)	138.27 (2.71)	139.28 (5.49)	139.82 (2.11)	141.67 (1.53)	138.72 (3.36)	138.87 (2.77)	138.85 (3.04)	0.867
K mmol/l (3.6–5.5)	4.14 (0.42)	4.19 (0.42)	4.19 (0.62)	4.19 (0.50)	4.30 (0.55)	4.33 (0.57)	4.20 (0.47)	4.50 (0.70)	4.00 (0.01)	0.948
Cl mmol/l (98–112)	102.84 (4.55)	103.63 (4.37)	101.93 (5.86)	104.19 (3.65)	104.32 (3.79)	105.33 (5.77)	103.68 (4.28)	104.43 (2.87)	103.38 (4.11)	0.164
Ca mmol/l (8.4–10.2)	9.01 (0.57)	9.18 (0.60)	9.12 (0.63)	10.97 (0.91)	9.43 (0.33)	8.97 (0.51)	8.92 (0.65)	8.97 (0.36)	9.05 (0.80)	*p * **<0** **.001**
Uric acid mg/dl (2.6–6.0)	5.00 (1.69)	5.45 (1.60)	6.22 (2.69)	5.17 (2.17)	6.00 (1.70)	5.40 (0.35)	4.89 (2.04)	5.73 (1.77)	5.57 (1.97)	0.119
Bilirub mg/dl (0.30–1.20)	0.68 (0.33)	0.70 (0.40)	0.61 (0.51)	0.71 (0.58)	0.65 (0.37)	0.82 (0.07)	0.71 (1.53)	0.58 (0.38)	0.68 (0.34)	0.855
AST I.U./l (7–35)	17.33 (5.38)	21.60 (12.13)	23.13 (29.62)	19.79 (13.74)	19.00 (10.42)	14.67 (2.31)	21.79 (19.44)	20.29 (15.11)	21.94 (14.30)	0.702
ALT I.U./l (7–55)	14.65 (7.06)	21.32 (17.10)	18.20 (16.02)	17.51 (15.40)	19.22 (14.02)	10.33 (6.66)	18.51 (24.66)	17.00 (9.96)	23.73 (18.19)	0.577
gGT U/l (5–35)	25.26 (27.61)	27.83 (23.11)	29.50 (17.97)	31.37 (38.72)	27.88 (22.64)	29.67 (15.89)	41.80 (55.37)	48.47 (44.48)	44.00 ± 39.2776	0.144
GLIC mg/dl (70–110)	121.58 (68.94)	114.57 (33.91)	110.67 (34.92)	105.59 (35.11)	106.04 (28.64)	103.00 (33.45)	100.70 (48.08)	117.35 (35.55)	112.57 (37.06)	0.067
ESR mm/h (<15)	40.38 (28.12)	45.31 (26.38)	54.27 (38.95)	39.78 (29.16)	61.09 (30.48)	52.00 (28.69)	56.97 (33.44)	59.52 (28.83)	52.31 (28.55)	*p * **<0** **.001**
CRP mg/dl (<0.50)	1.62 (2.66)	1.18 (2.73)	1.98 (3.02)	1.10 (2.24)	1.50 (2.24)	0.83 (0.97)	1.62 (2.57)	2.22 (4.22)	2.34 (3.59)	0.22
Folic acid ng/mL (4.6–18.7)	9.97 (15.35)	20.38 (76.10)	8.61 (11.96)	10.86 (23.44)	8.94 (8.81)	13.37 (7.58)	11.59 (18.87)	11.71 (11.84)	12.33 (18.44)	0.749
Vit B12 pg/mL (197–771)	457.42 (614.25)	429.17 (214.13)	630.00 (446.52)	512.04 (900.44)	830.50 (1455.45)	413.00 (202.30)	632.60 (1289.47)	544.92 (555.12)	625.57 (595.10)	0.803
Homoc micromol/l (5-12)	18.99 (7.99)	19.22 (7.16)	19.56 (5.95)	20.53 (11.12)	20.38 (12.12)	17.43 (2.47)	19.05 (8.56)	18.93 (8.98)	18.67 (5.22)	0.961
*Nutritional assessment*										
BMI kg/m^2^ (18.5–24.9)	24.90 (2.81)	30.44 (4.28)	21.25 (2.89)	25.25 (4.76)	23.96 (2.89)	27.68 (3.99)	19.59 (2.60)	24.786 (2.44)	25.343 (3.24)	*p * **<0** **.001**
*Strength*										
Handgrip dx kg (>20–>30)	22.14 (7.14)	17.71 (5.42)	17.64 (8.00)	16.51 (6.71)	17.47 (6.40)	22.67 (7.51)	16.02 (6.79)	14.00 (5.42)	18.06 (8.95)	*p * **<0** **.05**
*Risk of fractures*										
Frax anca %	10.48 (0.97)	10.83 (1.10)	10.09 (0.72)	7.75 (1.37)	10.48 (1.26)	10.77 (2.67)	10.57 (3.48)	10.071 (2.66)	15.63 (4.40)	*p * **<0** **.001**
*Hdration*										
TBW % (56–60)	54.76 (6.59)	48.43 (5.87)	60.08 (5.59)	52.26 (6.90)	52.66 (5.33)	50.71 (1.46)	58.44 (6.52)	48.73 (3.14)	51.42 (5.50)	*p * **<0** **.001**
ECW % (42–50)	49.93 (5.78)	51.73 (4.39)	52.99 (5.50)	53.15 (6.97)	52.66 (3.57)	55.97 (4.49)	55.53 (7.91)	55.00 (3.51)	51.25 (1.51)	*p * **<0** **.05**
ICW % (50–58)	50.05 (5.75)	48.29 (4.42)	46.57 (5.15)	46.77 (6.53)	47.52 (3.41)	44.03 (4.49)	44.54 (7.76)	45.00 (3.23)	48.75 (6.95)	*p * **<0** **.05**

**Table 3:** Association between VAT and SAT with other outcomes in osteosarcopenic obesity cohort.

Variables	Visceral adipose tissue (*r*)	Subcutaneous adipose tissue (*r*)
WBC K/*µ*L (4.00–10.00)	0.108 (*p*=0.442)	0.051 (*p*=0.719)
RBC M/*µ*L (4.00–5.00)	0.087 (*p*=0.540)	0.051 (*p*=0.719)
Hb g/dl (12.0–16.0)	0.063 (*p*=0.642)	0.003 (*p*=0.983)
Hct % (36.0–46.0)	0.019 (*p*=0.896)	0.020 (*p*=0.890)
MCV fL (81.0–93.0)	−0.723 (*p*=0.123)	−0.102 (*p*=0.722)
PLT K/*µ*L (140–450)	−0.155 (*p*=0.268)	**0.296 (** *p * **<0** **.05** **)**
Lymph *n* K/*µ*L (0.80–3.60)	−0.115 (*p*=0.518)	0.313 (*p*=0.069)
Iron mcg/dl (45–145)	−0.058 (*p*=0.782)	0.184 (*p*=0.200)
Trg mmol/L (<200)	−0.155 (*p*=0.255)	0.219 (*p*=0.222)
Total Chol mmol/L (<200)	−0.236 (*p*=.0.089)	0.238 (*p*=0.092)
Alb g/dl (4.02–4.76)	0.055 (*p*=0.692)	0.699 (*p*=0.153)
Creat mg/dl (0.57–1.11)	0.185 (*p*=0.189)	**−0.299 (** *p * **<0** **.05** **)**
Azotemia mg/dl (14–40)	0.190 (*p*=0.173)	−0.237 (*p*=0.94)
Na mmol/l (136–145)	−0.182 (*p*=0.192)	0.186 (*p*=0.192)
K mmol/l (3.6–5.5)	−0.574 (*p*=0.234)	**0.960 (** *p * **<0** **.01** **)**
Cl mmol/l (98–112)	0.087 (*p*=0.538)	0.126 (*p*=0.384)
Ca mmol/l (8.4–10.2)	−0.210 (*p*=0.130)	−0.022 (*p*=0.978)
Uric acid mg/dl (2.6–6.0)	−0.042 (*p*=0.765)	−0.048 (*p*=0.742)
Bilirub mg/dl (0.30–1.20)	**0.328 (** *p * **<0** **.05** **)**	−0.108 (*p*=0.457)
AST I.U./l (7–35)	0.076 (*p*=0.588)	−0.170 (*p*=0.233)
ALT I.U./l (7–55)	**0.340 (** *p * **<0** **.05** **)**	−0.130 (*p*=0.363)
gGT U/l (5–35)	−0.056 (*p*=0.696)	0.124 (*p*=0.385)
GLIC mg/dl (70–110)	0.049 (0 = 0.730)	0.101 (*p*=0.483)
ESR mm/h (<15)	−0.053 (*p*=0.706)	−0.031 (*p*=0.828)
CRP mg/dl (<0.50)	0.047 (*p*=0.742)	−0.050 (*p*=0.725)
Folic acid ng/mL (4.6–18.7)	0.128 (*p*=0.471)	0.032 (*p*=0.859)
Vit B12 pg/mL (197–771)	0.001 (*p*=0.944)	−0.065 (*p*=0.713)
Homoc micromol/l (5–12)	−0.098 (*p*=0.588)	−0.150 (*p*=0.413)
BMI kg/m^2^ (18.5–24.9)	**0.593 (** *p * **<0** **.001** **)**	**0.387 (** *p * **<0** **.01** **)**
FRAX anca %	**0.316 (** *p * **<0** **.05** **)**	0.050 (*p*=0.750)
Handgrip strength dx	0.335 (*p*=0.053)	−0.079 (*p*=0.668)
TBW % (56–60)	−0.180 (*p*=0.325)	−0.185 (*p*=0.312)
ECW % (42–50)	−0.274 (*p*=0.129)	0.219 (*p*=0.228)
ICW % (50–58)	−0.042 (*p*=0.767)	0.031 (*p*=0.867)

NS: not statistically significant.
